# Caffeine use disorder and analgesic dependence in medication-overuse headache: insights into an underrecognized relationship

**DOI:** 10.3389/fneur.2026.1827585

**Published:** 2026-07-13

**Authors:** Rehab Magdy, Amr Hassan, Mona Hussein, Alaa Elmazny, Osama Yacoub, Mohamed Abdelghaffar, Nourhan Abdelmohsen Taha, Ahmed Essmat, May M. Fayez, Nahla Merghany, Anas Elgenidi, Ahmed Dahshan

**Affiliations:** 1 Department of Neurology, Cairo University, Cairo, Egypt; 2 Department of Neurology, Beni-Suef University, Beni-Suef, Egypt; 3 College of Medicine and Health Sciences, Arabian Gulf University, Manama, Bahrain; 4 Department of Neurology, Fayoum University, Fayoum, Egypt; 5 Department of Neurology, Ain Shams University, Cairo, Egypt; 6 Department of Neurology, Al-Azhar University, Cairo, Egypt; 7 Department of Neurology, El Mounira Hospital, Cairo, Egypt; 8 Department of Neurology, Mayo Clinic, Jacksonville, FL, United States

**Keywords:** analgesic dependence, caffeine consumption, caffeine use disorder, medication-overuse headache, primary headache disorders

## Abstract

**Background:**

Caffeine plays a dual role in headache disorders, acting as an analgesic adjuvant while potentially contributing to headache chronification. Its relationship with medication-overuse headache (MOH) and the severity of analgesic dependence remains insufficiently characterized. The study aimed to compare MOH and non-MOH patients in terms of headache burden, psychological characteristics, and caffeine use, and secondarily to assess whether caffeine use disorder is associated with severity of dependence on analgesics in MOH.

**Methods:**

This is a cross-sectional study conducted on adults with primary headache disorders. Participants were asked to complete the following validated questionnaires: caffeine food frequency (C-FFQ), caffeine use disorder (CUDQ), headache impact test-6 (HIT-6), Severity of Dependence Scale (SDS). Depression Anxiety Stress Scales–12 (DASS-12), and Substance Use Risk Profile Scale (SURPS). Headache diaries were reviewed to document monthly headache days (MHD) and acute medication days (AMD); subsequently, patients were divided into MOH or non-MOH groups, according to ICHD-3 criteria.

**Results:**

Among 482 patients with primary headache disorders, 160 (33.2%) fulfilled ICHD-3 criteria for MOH. Patients with MOH had significantly higher MHD, AMD, HIT-6 scores, DASS-12 and SURPS total scores than those with non-MOH (all *p* < 0.001). Heavy caffeine consumption was more prevalent in MOH patients (23.8% vs. 9.3%), who also demonstrated higher CUDQ scores (median 10 vs. 7, *p* < 0.001). CUDQ scores correlated positively with MHD, AMD, HIT-6, DASS-12, and SURPS (all p < 0.001). Among patients with MOH, SDS scores correlated with MHD, AMD, HIT-6, DASS-12, SURPS, and CUDQ scores (all *p* < 0.05). Multivariable regression analysis revealed that AMD, HIT-6 score, and CUDQ score were independently associated with SDS scores (*p*-value = 0.002, 0.046, 0.002, respectively).

**Conclusion:**

Patients with MOH had significantly greater headache burden, psychological distress, and caffeine consumption compared with those without MOH. Caffeine use disorder is significantly associated with greater severity of analgesic dependence in patients with MOH. These findings highlight the importance of assessing caffeine-related behaviors when evaluating patients with MOH and suggest that caffeine use disorder may represent a clinically relevant behavioral factor associated with medication dependence severity.

## Introduction

Caffeine has a complex relationship with headaches. It is well established that caffeine exerts an antinociceptive effect primarily by antagonizing adenosine receptors (1). It also acts as an adjuvant to other analgesics; adding caffeine to acetaminophen reduces the dose required to achieve the same effect by 40% (2). On the contrary, long-term excessive caffeine consumption may increase the risk of transformation of primary headache to chronic disorders (3, 4). Such a double player effect of caffeine is best recognized with migraine, where it can relieve acute migraine attacks and act as a trigger for the attacks (5).

Another double player effect of caffeine is assumed with medication overuse headache (MOH). Since excessive caffeine intake can worsen primary headaches, it may lead the patient to take painkillers too frequently, which simply in turn may lead to MOH. From another perspective, caffeine itself is considered a pain reliever, so does excessive consumption of it specifically cause MOH. Espinosa Jovel and Sobrino Mejía (6) supposed that long-term excessive caffeine consumption increases cortical hyperexcitability and promotes the release of pro-nociceptive neuropeptides, leading to MOH. Yet, caffeine was not listed by the International Headache Society as a substance that potentially causes MOH (7).

Another link is based on the classification of caffeine as a substance with potential dependence (8). On the other hand, various studies indicated that patients with MOH may have underlying dependency-related behavior (9). Such an association could indicate a potential causal role.

In addition, the circumstances that inspire a patient to develop substance dependency, whether on caffeine or painkillers, are intermingled with depression, anxiety, and stress (10, 11). All these psychiatric comorbidities are well-known risk factors of developing MOH (12, 13).

Based on this background, the primary objective of the current study was to compare headache burden, psychological characteristics, caffeine consumption, and caffeine use disorder between patients with and without MOH. The secondary objective was to explore the relationship between caffeine use disorder and the severity of dependence on acute headache medications among patients with MOH.

## Methods

### Study design and eligibility criteria

This cross-sectional study was conducted between February 2025 and October 2025, on patients ≥18 years diagnosed with primary headache disorders in accordance with the International Classification of Headache Disorders, 3rd edition (ICHD-3) (14). The patients were recruited from four specialized headache clinics at Cairo, Ain Shams, Fayoum, and Al-Azhar University Hospitals, Egypt. Patients with secondary headache disorders, co-morbid chronic pain disorder, alcohol or illicit substance use disorders, severe psychiatric or cognitive impairment were excluded.

### Data collection

Contact information for 800 patients with a confirmed diagnosis of primary headache disorders was obtained from the medical records of the four participating headache centers. Those patients were invited to participate in the present study via WhatsApp messaging, which was selected because it is a widely accessible communication platform. The invitation included a concise explanation of the study objectives, design, and procedures, as well as an electronic informed consent form.

A validated caffeine food frequency questionnaire (C-FFQ) (15) was sent to the patients to assess their daily consumption of common caffeine-containing sources. Participants were instructed to prospectively complete this questionnaire and also to record monthly headache days (MHD) and acute medication days (AMD) over the upcoming month using a standardized headache diary, which was used solely to document these two variables. Participants were informed that, upon completion of the recording phase (1 month), they would be invited to attend a face-to-face interview at their respective headache center for a comprehensive clinical assessment of their headache and other relevant clinical variables.

### Clinical assessment

Out of 800 invited patients, only 482 accepted the invitation and attended their respective headache centers for clinical assessment. During the face-to-face interview, the diagnosis of medication-overuse headache (MOH) was established in accordance with the ICHD-3 criteria (14). Headache diaries were reviewed to verify the recorded MHD and AMD, and the C-FFQ was checked for completeness and consistency. Based on the estimated daily caffeine intake, participants were categorized as heavy (≥400 mg/day), light (<400 mg/day), or non-caffeine consumers.

Participants were also asked about their reasons for consuming caffeine. Additionally, they were asked about the perceived effects of caffeine on their headaches, specifically whether they believe it alleviates headache or acts as a potential trigger or exacerbating factor for it.

Furthermore, problematic patterns of caffeine use were assessed using the Caffeine Use Disorder Questionnaire (CUDQ) (16), a brief self-report measure assessing DSM-5–based symptoms of problematic caffeine use, with higher scores indicating greater symptom severity. The scale is typically scored using a Likert format, with total scores ranging from 0 to 40.

During the same visit, participants were asked to complete additional standardized self-administered questionnaires. Headache-related disability was assessed using the Headache Impact Test–6 (HIT-6) (17), a six-item questionnaire measuring the impact of headaches on daily functioning, with total scores ranging from 36 to 78, where higher scores indicate greater disability.

Dependence related to acute headache medication use was evaluated using the Severity of Dependence Scale (SDS) (18), a five-item instrument assessing psychological dependence on analgesics, with total scores ranging from 0 to 15, and higher scores reflecting greater dependence severity.

Psychological distress was measured using the Depression Anxiety Stress Scales–12 (DASS-12) (19), which assesses symptoms of depression, anxiety, and stress experienced over the preceding month, yielding subscale scores ranging from 0 to 12.

Personality traits associated with vulnerability to substance use were assessed using the Substance Use Risk Profile Scale (SURPS) (20), a 23-item questionnaire measuring four stable personality traits, hopelessness, anxiety sensitivity, impulsivity, and sensation seeking, analyzed as continuous variables without diagnostic cut-off values.

### Primary outcome

To compare headache burden, psychological characteristics, caffeine consumption, and caffeine use disorder between patients with and without MOH.

### Key secondary outcome

To evaluate factors associated with the severity of medication dependence, assessed using the SDS, among patients with MOH, with particular focus on the association between caffeine use disorder and dependence severity.

### Sample size

The sample size was calculated using EpiCalc 2000, version 1.02, 1997. Based on an alpha level of significance of 0.05, 51.4% prevalence rate of primary headache disorders in Fayoum Governorate in Egypt (21), a null hypothesis of 45%, a total sample size of at least 475 participants was required to achieve a statistical power of 80.0%.

### Statistical methods

Data were analyzed using SPSS version 25 (IBM Corp., Armonk, NY, United States). The Kolmogorov–Smirnov test was used to test the normality of data. Quantitative data such as age, BMI, duration of headache disorder, AMD, MHD, SDS, HIT-6, SURPS, DASS-12, and CUDQ were expressed as median and interquartile range. Categorical data such as sex, type of headache, caffeine consumption and beliefs about the relationship between headaches and caffeine consumption were expressed as number and percentage. Mann–Whitney U-test was used to compare between patients with and without MOH in quantitative data, while chi-square test was used to compare between the two groups in categorical data. Spearman correlation was used to correlate CUDQ and SDS with age, BMI, disease duration, AMD, MHD, HIT-6, SURPS, and DASS-12. Multiple linear regression analysis was done to identify factors associated with SDS scores after being adjusted for their potential mutual confounding effect. The independent variables were determined based on their clinical relevance. The *p*-values were adjusted for multiple testing using Benjamini and Hochberg procedure. All tests were two-tailed.

## Results

### Demographic and clinical characteristics of the included patients

This cross-sectional study was conducted on 482 patients with primary headache disorders (160 with MOH and 322 without MOH). There were no statistically significant differences between patients with MOH and those without MOH regarding age (*p*-value = 0.174), sex distribution (*p*-value = 0.255), BMI (*p*-value = 0.363), type or duration of primary headache disorder (*p*-value = 0.875, 0.361 respectively). In contrast, patients with MOH had significantly higher MHD (median 20 vs. 7, *p*-value < 0.001), AMD (median 18 vs. 4, *p*-value < 0.001), and HIT-6 total scores (median 65 vs. 60, *p*-value < 0.001) compared with patients without MOH (Table 1).

**Table 1:** Demographics and clinical characteristics of the included patients.

Characteristic		Patients with MOH (*n* = 160)	Patients without MOH (*n* = 322)	*P*-value
Age [median (IQR)]		34 (27–40)	35 (28.75–41)	0.174
Sex [*n* (%)]	Males	59 (36.9%)	102 (31.7%)	0.255
	Females	101 (63.1%)	220 (68.3%)	
BMI [median (IQR)]		27.34 (24.4–30.85)	26.9 (24.15–30.48)	0.363
Type of primary headache disorder [*n* (%)]	Tension	51 (31.9%)	96 (29.8%)	0.875
	Migraine	97 (60.6%)	203 (63.0%)	
	Cluster	12 (7.5%)	23 (7.1%)	
Duration of primary headache disorder [median (IQR)]		3 (2–8)	4 (2–8)	0.361
Duration of MOH [median (IQR)]		1 (0.5–3)	–	–
MHD [median (IQR)]		20 (15–30)	7 (4–10)	<0.001*
AMD [median (IQR)]		18 (15–25)	4 (2–7)	<0.001*
HIT-6 total score [median (IQR)]		65 (62–70)	60 (56–64)	<0.001*
SDS total score [median (IQR)]		9 (7–11.75)	–	–
DASS-12 [median (IQR)]	DASS-D (depression)	4 (2–8)	3 (1–4)	<0.001*
	DASS-A (anxiety)	6 (3–8)	4 (2–6)	<0.001*
	DASS-S (stress)	7 (4–9)	4 (3–7)	<0.001*
	Total score	18 (11–24)	12 (6–17)	<0.001*
SURPS [median (IQR)]	Hopelessness	15 (12–19)	14 (12–16)	0.002*
	Anxiety sensitivity	15 (13–16)	14 (12–15)	0.060
	Impulsivity	13 (10–16)	12 (10–14)	<0.001*
	Sensation seeking	13 (11–16)	13 (10–15)	0.017*
	Total score	56 (49.25–64)	53 (46–58.25)	<0.001*
Caffeine consumption [n (%)]	Non-consumers	33 (20.6%)	53 (16.5%)	<0.001*
	Light consumers	89 (55.6%)	239 (74.2%)	
	Heavy consumers	38 (23.8%)	30 (9.3%)	
CUDQ total score [median (IQR)]		10 (4–18)	7 (2–10)	<0.001*

AMD, acute medication days; BMI, body mass index; CUDQ, Caffeine Use Disorder Questionnaire; DASS-12, Depression Anxiety Stress Scales–12; HIT-6, Headache Impact Test–6; MHD, monthly headache days; MOH, medication overuse headache; SDS, Severity of Dependence Scale; SURPS, Substance Use Risk Profile Scale.

*p-value < 0.05 is considered significant.

### Psychological and behavioral measures

Patients with MOH demonstrated significantly higher DASS-12 depression scores (median 4 vs. 3, *p*-value < 0.001), anxiety scores (median 6 vs. 4, *p*-value < 0.001), stress scores (median 7 vs. 4, *p*-value < 0.001), and total DASS-12 scores (median 18 vs. 12, *p*-value < 0.001) compared with patients without MOH (Table 1).

Regarding SURPS scale, patients with MOH showed significantly higher hopelessness scores (median 15 vs. 14, *p*-value = 0.002), impulsivity scores (median 13 vs. 12, *p*-value < 0.001), sensation-seeking scores (median 13 vs. 13, p-value = 0.017), and total SURPS scores (median 56 vs. 53, *p*-value < 0.001) compared with patients without MOH. Anxiety sensitivity scores did not differ significantly between groups (*p*-value = 0.060) (Table 1).

### Caffeine consumption patterns and patients’ beliefs about the relationship between caffeine and headache

Caffeine consumption differed significantly between patients with MOH and those without MOH (*p*-value < 0.001). Heavy caffeine consumption was significantly higher among patients with MOH (23.8%) than among those without MOH (9.3%), whereas light caffeine consumption was more common in patients without MOH (74.2% vs. 55.6%). Patients with MOH also had significantly higher CUDQ scores than patients without MOH (median 10 vs. 7, *p*-value < 0.001) (Table 1).

Among caffeine consumers (*n* = 396), 80.1% reported consuming caffeine to improve alertness or energy, 66.2% for flavor or enjoyment, 57.8% to relieve headache, and 38.4% consume it as a habit. Only 30.3% of participants reported that caffeine consistently improved their headache, while 37.6% reported occasional improvement. In contrast, 5.1% reported that caffeine worsened headache, and 19.2% reported that it sometimes triggered headache, whereas 21.2% were uncertain about its effect (Table 2).

**Table 2:** Beliefs about the relationship between caffeine consumption and headache.

Question	Response	Caffeine consumers (*n* = 396)
Why do you consume caffeine?	To relieve headache	229 (57.8%)
	To improve alertness/energy	317 (80.1%)
	Habit	152 (38.4%)
	Flavor/enjoyment	262 (66.2)
Do you believe that caffeine improves your headache?	Yes	120 (30.3%)
	Sometimes	149 (37.6%)
	No	88 (22.2%)
	I do not know	39 (9.8%)
Do you believe that caffeine makes your headache worse or triggers headaches?	Yes	20 (5.1%)
	Sometimes	76 (19.2%)
	No	216 (54.5%)
	I do not know	84 (21.2%)

### Correlations between caffeine use disorder and clinical variables

CUDQ scores showed a weak but significant negative correlation with duration of the primary headache disorder (*r* = −0.108, *p*-value = 0.032). Significant positive correlations were observed between CUDQ total scores and MHD (*r* = 0.192, *p*-value < 0.001), AMD (*r* = 0.233, *p*-value < 0.001), HIT-6 total scores (*r* = 0.201, *p*-value < 0.001), DASS-12 total scores (*r* = 0.467, *p*-value < 0.001), and SURPS total scores (*r* = 0.438, *p*-value < 0.001) (Table 3).

**Table 3:** Correlations between CUDQ in caffeine consumers and their demographics and clinical characteristics.

Characteristic	CUDQ	
	(r) Coef.	*P*-value
Age	−0.012	0.809
BMI	−0.006	0.909
Duration of primary headache disorder	−0.108	0.032*
MHD	0.192	<0.001*
AMD	0.233	<0.001*
HIT-6 total score	0.201	<0.001*
DASS-12 total score	0.467	<0.001*
SURPS total score	0.438	<0.001*

AMD, acute medication days; BMI, body mass index; CUDQ, Caffeine Use Disorder Questionnaire; DASS-12, Depression Anxiety Stress Scales–12; HIT-6, Headache Impact Test-6; MHD, monthly headache days; SURPS, Substance Use Risk Profile Scale.

(*r*) Coef., Spearman correlation coefficient.

The *p*-values were adjusted for multiple testing using Benjamini and Hochberg procedure.

*p-value ≤ 0.032 is considered significant.

### Correlations between severity of dependence on medications in MOH and clinical variables

In patients with MOH, SDS scores were significantly correlated with MHD (*r* = 0.173, *p*-value = 0.029), AMD (*r* = 0.396, *p*-value < 0.001), HIT-6 total scores (*r* = 0.328, *p*-value < 0.001), DASS-12 total scores (*r* = 0.265, *p*-value = 0.001), SURPS total scores (*r* = 0.264, *p*-value = 0.001), and CUDQ total scores (*r* = 0.299, *p*-value = 0.001) (Table 4).

**Table 4:** Correlations between SDS in patients with MOH and their demographics and clinical characteristics.

Characteristic	SDS	
	(*r*) Coef.	*P*-value
Age	−0.018	0.818
BMI	−0.111	0.161
Duration of MOH	0.133	0.093
MHD	0.173	0.029*
AMD	0.396	<0.001*
HIT-6 total score	0.328	<0.001*
DASS-12 total score	0.265	0.001*
SURPS total score	0.264	0.001*
CUDQ total score	0.299	0.001*

AMD, acute medication days; BMI, body mass index; CUDQ, Caffeine Use Disorder Questionnaire; DASS-12, Depression Anxiety Stress Scales–12; HIT-6, Headache Impact Test–6; MHD, monthly headache days; MOH, medication overuse headache; SDS, Severity of Dependence Scale; SURPS, Substance Use Risk Profile Scale.

(*r*) Coef., Spearman correlation coefficient.

The *p*-values were adjusted for multiple testing using Benjamini and Hochberg procedure.

*p-value ≤ 0.029 is considered significant.

### Factors associated with severity of dependence on medications in MOH

Multiple linear regression analysis was done to identify factors associated with SDS scores. The selection of independent variables was based on their clinical relevance. Therefore, the following variables were included in the model: age, female sex, BMI, duration of MOH, tension headache, cluster, MHD, AMD, HIT-6 total score, DASS-12 total score, SURPS total score, heavy caffeine consumption, and CUDQ total score. Multicollinearity was evaluated using tolerance and VIF values. All tolerance values were above 0.2, and all VIF values were below 5, indicating that the independent variables were not highly correlated. Adjusted *R*^2^ was 0.236.

The model revealed that AMD, HIT-6 total score, and CUDQ total score were independently associated with SDS scores (*p*-value = 0.002, 0.046, 0.002, respectively) (Table 5).

**Table 5:** Factors associated with SDS in patients with MOH.

Characteristic		B	*P*-value	95.0% CI for B		Collinearity statistics	
				Lower bound	Upper bound	Tolerance	VIF
(Constant)		−2.511	0.487	−9.644	4.622		
Age		0.037	0.239	−0.025	0.098	0.715	1.399
Female sex		0.384	0.527	−0.816	1.584	0.628	1.591
BMI		−0.015	0.758	−0.111	0.081	0.782	1.279
Duration of MOH		0.020	0.844	−0.181	0.221	0.855	1.170
Type of primary headache disorder	Tension-type headache	−1.112	0.063	−2.287	0.063	0.759	1.317
	Cluster headache	−1.385	0.163	−3.337	0.567	0.740	1.352
MHD		−0.091	0.199	−0.231	0.049	0.273	3.664
AMD		0.254	0.002*	0.092	0.416	0.240	4.173
HIT-6 total score		0.086	0.046*	0.002	0.170	0.732	1.366
DASS-12 total score		−0.071	0.075	−0.148	0.007	0.435	2.301
SURPS total score		0.031	0.368	−0.037	0.100	0.448	2.234
Heavy caffeine consumption		−0.460	0.544	−1.955	1.035	0.476	2.102
CUDQ total score		0.160	0.002*	0.060	0.260	0.369	2.711

AMD, acute medication days; BMI, body mass index; CUDQ, Caffeine Use Disorder Questionnaire; DASS-12, Depression Anxiety Stress Scales–12; HIT-6, Headache Impact Test–6; MHD, monthly headache days; MOH, medication overuse headache; SDS, Severity of Dependence Scale; SURPS, Substance Use Risk Profile Scale.

Adjusted *R* square = 0.236.

*P-value < 0.05 is considered significant.

### Medication use, dependence severity, psychological characteristics, and caffeine use disorder across primary headache subtypes (MOH and non-MOH groups)

There were no significant differences between primary headache subtypes regarding AMD, SDS total scores, DASS-12 total scores, or SURPS total scores (all *p*-values > 0.05). In contrast, CUDQ scores differed significantly across headache groups (*p* = 0.002). Patients with tension-type headache had significantly higher scores compared with those with migraine (*p* = 0.003). Cluster headache patients also showed significantly higher scores than migraine (*p* = 0.012), but their scores did not differ significantly from those with tension-type headache (*p* = 0.423). These findings suggest that problematic caffeine use is more pronounced in tension-type and cluster headache groups compared with migraine (Table 6).

**Table 6:** Medication use, dependence severity, psychological characteristics, and caffeine use disorder across primary headache subtypes (MOH and non-MOH groups).

Characteristic	Migraine group^1^ (*n* = 300)	Tension-type headache group^1^ (*n* = 147)	Cluster headache^1^ group (*n* = 35)	*p*-value
AMD	7 (3–15)	7 (3–15)	10 (4–20)	0.311
SDS total score	9 (7–12)	9 (7–10)	9 (7–12)	0.116
DASS-12 total score	12 (7–19)	12 (8–20)	15 (8–21)	0.272
SURPS total score	54 (47–60)	54 (47–61)	55 (45–62)	0.835
CUDQ total score	6 (2–11)	10 (5–15)	10 (5–17)	0.002*

AMD, acute medication days; CUDQ, Caffeine Use Disorder Questionnaire; DASS-12, Depression Anxiety Stress Scales–12; SDS, Severity of Dependence Scale; SURPS, Substance Use Risk Profile Scale.

^1^Participant counts represent the full study sample across both MOH and non-MOH groups.

*p-value < 0.05 is considered significant.

## Discussion

Overall, this study identified significant differences between patients with MOH and those without MOH, with MOH patients demonstrating greater headache burden, higher acute medication use, increased psychological distress, and different caffeine-related behaviors.

The prevalence of MOH in our study was 33.2%, consistent with the well-recognized variation across clinical settings: roughly 1–2% in the general population versus 30–70% in specialized headache centers (22). This gradient largely reflects referral filtering, as patients with more frequent, disabling, and treatment-refractory headaches are disproportionately seen in specialty care. Because our patients were recruited from such clinics, the observed prevalence should be read in that light and may not generalize to community-based populations with primary headache disorders.

In the present study, patients with MOH had significantly higher depression, anxiety, and stress scores of DASS-12 than those without MOH. This finding was in line with previous studies, even when different assessment tools were used (23, 24). Serotonergic disturbances implicated in depression and anxiety disorders may play a role in the pathophysiology of MOH (25). Furthermore, underlying anxiety and depression may alter pain-coping strategies and encourage the patient toward frequent use of analgesics (26). Hagen et al. (27) found that a score of ≥11 on the Hospital Anxiety and Depression Scale increased the risk of developing MOH by 5 times. In our study, although DASS-12 scores were significantly correlated with SDS scores, psychological distress did not remain an independent predictor of medication dependence severity among patients with MOH after multivariable adjustment. This difference may reflect the overlapping contribution of psychological distress with other behavioral and clinical factors, such as caffeine use disorder, personality traits, and medication use patterns, which may better explain the variability in dependence severity.

From the behavioral perspective assessed by SURPS, patients with MOH showed significantly higher hopelessness, impulsivity, and sensation-seeking scores compared with patients without MOH. Correspondingly, a large sample of patients with MOH (*n* = 895) was screened for DSM-IV criteria for substance dependence by Fuh et al. (28), by whom 68% fulfilled the criteria. The pathophysiological mechanism of addictive behavior involves dysfunction of the orbitofrontal cortex (OFC), leading to loss of its inhibitory control over compulsive drug-seeking behavior (29). Intriguingly, microstructural changes in the OFC are demonstrated in patients with MOH (30). Moreover, shared susceptibility genes between MOH and drug dependence have been shown convincingly (31). Notably, SURPS anxiety sensitivity was the only subscale that did not differ significantly between MOH and non-MOH groups (*p* = 0.060). This may be explained by individuals with high anxiety sensitivity having an increased fear of the physical and psychological symptoms of anxiety, and are therefore less likely to engage in heavy substance abuse (32).

Recent evidence suggests that caffeine use disorder may be more than a marker of excessive intake, instead reflecting an underlying susceptibility to dependence-related behaviors that may be associated with medication overuse (33). Shared neurobiological substrates of dependence, behavioral reinforcement, and addiction-related personality traits, notably impulsivity and sensation-seeking may underlie both caffeine misuse and excessive analgesic consumption (34, 35).

Therefore, the relationship between CUDQ and SDS should be interpreted cautiously because both instruments capture facets of compulsive use and loss of control, raising the possibility that their observed association may partly reflect common behavioral or personality-related susceptibility rather than two distinct clinical phenomena. Future studies using more rigorous psychometric approach, such as confirmatory factor analysis or latent variable modeling, would help determine whether caffeine use disorder represents an independent contributor to medication dependence or a marker of shared vulnerability.

Notably, although the regression model in the present study revealed that AMD, HIT-6 total score, and CUDQ total score were independently associated with SDS scores, the model’s overall explanatory power was modest (adjusted *R*^2^ = 0.236), leaving much of the variability in analgesic dependence severity unexplained. Additional genetic, biological, psychological, and behavioral factors not assessed here may also contribute substantially to dependence severity in patients with MOH (22).

The third notable difference was that heavy caffeine consumption was significantly higher among patients with MOH than those without MOH (23.8% versus 9.3%). On the contrary, light caffeine consumption was more common in patients without MOH (74.2% vs. 55.6%). These findings confirmed that the biological effects of caffeine are dose-dependent (36). Long-term excessive caffeine consumption leads to overregulation and hypersensitivity of adenosine receptors, which mediate abnormally enhanced glutamatergic signaling and, eventually, a state of neuronal excitability. Moreover, prolonged activation of A2A receptors mediates the effects of calcitonin gene-related peptide (CGRP), a powerful pronociceptive neuropeptide (6).

Patients’ perceptions of caffeine’s effect on headache were notably heterogeneous in the present study. Although more than half of caffeine consumers used it specifically to relieve headache, only a minority reported consistent improvement, while the remainder described occasional benefit, worsening, triggering, or uncertainty. This pattern underscores the complex, bidirectional relationship between caffeine and headache (37). The gap between the frequent use of caffeine for relief and its inconsistent perceived benefit further suggests that factors beyond symptomatic efficacy, habitual consumption or dependence-related behaviors drive caffeine use in this population (38).

Ultimately, it cannot be definitively established whether excessive caffeine intake can provoke MOH in the context of this cross-sectional study. The current findings raise the following question to be answered by forthcoming research: Would attempting to stop/withdraw painkillers in patients with severe MOH be more successful and easier if they moderated their caffeine intake?

On the other hand, AMD and HIT-6 scores were independently associated with the SDS scores, whereas MOH duration was not, emphasizing that the severity of dependence is largely driven by headache burden and the frequency of overuse medications rather than how long the patient has taken these medications. The dependency-like behaviors exhibited by patients with MOH are primarily focused on pain-seeking relief rather than gaining euphoric effects. Consequently, SDS scores are associated with the biological need to reduce headache burden, not with the duration of overuse.

Critically, the current results must be interpreted cautiously due to the following limitations. First, due to the constraints of a cross-sectional design, the causal relationship between caffeine intake and MOH could not be definitely proven. Future prospective studies may further explore this aspect. An additional limitation is the absence of a non-response analysis. Only 482 of the 800 invited patients (60.3%) completed the study. Since detailed clinical and questionnaire data were available only for responders, we were unable to compare responders and non-responders. Therefore, the possibility of selection bias cannot be excluded, and the prevalence estimates of MOH and caffeine use disorder should be interpreted with caution.

Furthermore, participants were recruited from specialized headache clinics, where patients often present with more severe and complex headache disorders; therefore, the observed prevalence of MOH and the study findings may not be fully generalizable to all primary headache patients. Also, all participants were recruited from Egyptian hospitals, so the findings should be interpreted within Egypt’s cultural and healthcare context. Caffeine consumption patterns, attitudes toward medication use, and healthcare access may differ elsewhere. Generalizability to other populations and settings may therefore be limited, and validation in diverse international cohorts is warranted.

Moreover, the study measures relied on self-reported data rather than objective clinical markers. Although validated PROMs were used, objective verification of caffeine intake via biomarkers and of medication use was not performed. Therefore, recall, reporting, and social desirability biases cannot be entirely excluded. Also, the correlation between CUDQ and SDS may be inflated because both scales capture the same underlying psychological trait, e.g., tendency toward dependency, rather than two distinct physiological processes.

In addition, the SDS was administered only among patients with MOH, as the study focused on determining factors associated with the severity of medication dependence in individuals with established medication overuse. However, this approach does not allow assessment of dependence-related behaviors among non-MOH patients with frequent medication use, which should be explored in future studies. Finally, the C-FFQ was completed independently by participants prior to the clinical visit, whereas the remaining assessments were completed during the face-to-face evaluation, potentially introducing differential reporting bias.

## Conclusion

Patients with MOH exhibited significantly greater headache burden, psychological distress, and caffeine consumption compared with those without MOH. Notably, caffeine use disorder was associated with greater severity of medication dependence among patients with MOH. However, given the cross-sectional design of this study, the directionality of this association cannot be determined, and causal inferences should be avoided. These findings are hypothesis-generating and underscore the need for longitudinal research to clarify the temporal relationship between medication dependence and caffeine use disorder, and to identify patients at risk of severe drug dependence.

## Data Availability

The raw data supporting the conclusions of this article will be made available by the authors, without undue reservation.
